# On the Use of Water in Surgery

**Published:** 1851-08

**Authors:** Alphonse Auguste Amussat

**Affiliations:** Paris


ART. II.—ON THE USE OF WATER IN SURGERY. *By Alphonse Auguste Amussat, of Paris ; published at Paris, Jan.* 1851, *and translated from the French, by Frank H. Hamilton, Prof. of Surgery in the University of Buffalo.*

(*Continued from page* 89.)

IRRIGATIONS.

In surgical language, irrigation (from the word *irrigare*, to irrigate) is the bathing of a part; but thus considered, as M. Malgaigne has shown, the sense of this word is vague, for affusions, injections, *douches*, etc., are bathings, and according to this definition, ought to be called *irrigations*. I think it is more suitable, and conforms better with common usage, to reserve the word irrigation to designate the uniform flow of a liquid over the surface of the tissues.

Irrigations demanding special apparatus, I will pass in review successively those means which have been employed by the various surgeons who have given most attention to this subject, and I will explain finally an extemporaneous method to which we may resort when the appliances are not at hand for any of the ordinary modes.

*Apparatus of M. Josse.*

M. Josse describes as follows the apparatus which he has employed for irrigations:

A vessel having a spigot near its bottom, is filled with water, and placed upon a narrow and high table, at the bedside of the patient. The table should be about eighteen inches above the wounded limb. An oil cloth is laid so as to protect the bed, and facilitate the escape of the water into a pail placed below and into which the extremity of the oil cloth is made to extend. This apparatus may, however, be modified a thousand ways, and sometimes, doubtless, with advantage.

Two pails, which can always easily be obtained, may serve the same purpose, perhaps even more conveniently. One, having a spigot, or in which simply a hole has been made, is suspended above the bed; the other is placed beside the bed to receive the water as it flows off. That the direction of the current may be more easily changed, it is well to adjust to the spigot, or insert into the hole which has been made, a tube of some kind, and if possible, a gum elastic tube, the flexibility of which renders it very convenient. Even the oil cloth can be replaced by some other material; thus the ancients used the skin of an animal. This method might perhaps be revived advantageously, for while a prepared skin does not allow the water to filter through it, it imbibes, nevertheless, a certain amount of moisture which gives it a useful suppleness and coolness. We may also replace the oil cloth with a sheet of metal or of any other impermeable substance. But the best way to use such solid bodies is to form of them a gutter, in which the limb may be laid upon some soft body capable of absorbing moisture. But it should be especially borne in mind that the parts must not be kept exposed to the air.

Every thing being arranged, the diseased part is placed in the most convenient position; it is covered with compresses loosely laid upon it; another strip of cloth (*where the tube is not in use.*—T.) is secured at one end around the spigot, and the other reaches to the dressings, resting first upon that part of them which is the most elevated, and from thence extending downward along the limb. This is designed to prevent the water falling with its whole weight upon the diseased structures, and also to disperse it over a wider surface."

*M. Breschet's apparatus for irrigation.*

"We should have two large pails, one full of spring or well water, and the other empty. The latter is placed at the foot of the bed of the patient to receive the water which bathes the limb. We suspend the pail containing water to the tester of the bed, by a strong block or rope, or in any other way; we arrange it so that the bottom of the pail shall be directly over the wounded limb, and one or two feet above it. Then we must have a small glass or tin tube, of about the size of the little finger, bent at a very obtuse angle near its middle, so as to represent the letter U, with unequal branches of about three or four feet in length. We place one branch of this tube in the pail of water, and the other hangs over the wounded limb, the middle resting upon the edge of the pail. By a well known law of hydrostatics, the water may be immediately made to pass from the pail through the tube, and be thus conducted upon the diseased limb in a jet. In order, however, to set the current in motion, it is necessary to produce a momentary vacuum in the tube, by applying the mouth to the extremity of the outer branch. The water now passes in a continuous stream through the tube, upon the limb. But to secure all its possible advantages, two conditions are necessary. First — The water must not fall from a great height; and this is why it is important that the external branch should be very long, or the pail hung as low as possible. Second — The stream of water must be very fine and continuous; which may be effected by a small perforated cork adjusted to the outer extremity of the tube, or by what answers the purpose equally well, a bit of sponge introduced in the same manner. This water, after having impregnated the compresses, the bandages and the lint which envelopes the limb, passes to the limb itself, bathes it, and falls upon the oil cloth, to be conducted from the bed into the empty pail of which we have spoken. The surgeon or the attendant, has only to renew the water in the pail above as often as it becomes empty. The bandages and the dressings of the wound should be renewed every second or third day, or even less frequently, according to the judgment of the surgeon.

*Apparatus of A. Berard.*

The apparatus of A. Berard is like that of Breschet; except that in place of a single metallic syphon, the mouth of which is closed with a cork more or less fluted, he employs one or several tubes of glass of small diameter, according to the degree of refrigeration which he desires to produce.

M. Velpeau has figured in his *Operative Surgery* (vol. 1, p. 265 of the original, and vol. 1, p. 217 of the translation by Townsend.—T.) a pail furnished at its bottom with a spigot, which communicates with a horizontal tube of the length of the limb to be bathed, and from this tube spring six other small vertical tubes, which direct the water upon the surface of the limb.

*Apparatus of Mathias Mayor.*

" A vessel of some kind, appropriate to contain water and to be suspended above the patient, must be pierced with one or more little perforations, destined to receive the ends of pack thread smaller than the perforations. The vessel being now filled with water it will escape and be conveyed along these threads to the parts which we wish to irrigate, and upon which these conducting filaments have been laid.

" The property which fluids possess of coursing along a material so easily procured as pack thread, and the facility of adjusting these strings so as to enable us to regulate at will the quantity of water and its direction, these qualities, I say, are invaluable, and demand our consideration. But what is of still greater consequence, the water follows these threads wherever they are placed, even when they lie very obliquely. This liquid, in fact, does not easily leave its conductor, except when the direction of this conductor is nearly horizontal, or when it touches another object, or when there is a knot loosely tied in the course of the threads.

"In the second place, we can in this manner conduct the water from a distance, for however far the threads are stretched, they still continue to serve as aqueducts.

"A third fact to be noticed, is that the flow of water will be proportional to the size of the filiform conductor, the current being larger when the bundle of threads is larger, and the reverse.

"A fourth point of importance, is that the conductor is soft and flexible, and therefore is harmless if pushed against the sensitive parts, or if these are accidentally thrust against the apparatus.

"A fifth circumstance favorable to this plan, is that the liquid passes *imperceptibly* upon the part, and does not irritate by its fall; while a liquid cannot descend from tubes without a kind of shock being always felt at the place where it strikes the limb.

"A sixth consideration, and one which results directly from this absence of percussion on the part of the water, and from its gradual diffusion, as well as from the flexibility and suppleness of the conduit, is that the water will reach its destination even when there is some motion in the vessel or the threads, or in the part itself which is submitted to irrigation.

"We can easily distribute the water to several persons at the same time, and from the same vessel. This vessel is pierced with a number of holes, into which the ends of a corresponding number of threads are passed, and drawn through sufficiently to be secured to some point of the rim of the vessel. Each perforation not in use, is closed with a little plug, which may be removed at pleasure to permit the water to escape. To a like simple apparatus we can have recourse wherever the wounded are brought together in an ambulance, in a hospital, or in a carriage intended to transport one or more wounded."

M. Mayor has also proposed to replace the syphon by a fillet of coarse cloth.

*Apparatus of M. Guyot, Surgeon to the Hospital of Rennes.*

This apparatus for irrigation is composed of three boards and a large tunnel. The boards are placed as follows: Two short upright pieces are secured to either side of the bedstead, upon which the third board is laid transversely. This latter is perforated with a hole sufficiently large to receive the end of the tunnel, in which, when arranged, the tunnel is to be dropped, its mouth resting directly over the part to be irrigated. The oil cloth must be placed so as to protect the bed and drain off the water in the usual manner.*

M. Chaumette, of the hospital of Saint-Andre, at Bordeaux, employs also a large tunnel, which he suspends over the bed of the patient. From the lower orifice of the tunnel a bundle of pack thread reaches to the part which he wishes to irrigate.

*Apparatus of Dr. James Macartney.*

"This is a box made of zinc, much like a fracture box, in which the upper or lower extremities of the patient may be placed. The water, or the medicated fluids are conducted by means of a flattened tube, of which one extremity is joined to the reservoir, and the other traverses the upper wall of the box. This tube contains a band of coarse cloth, which is large at one end, and at the other terminates in a point. The first is received in the vessel which contains the liquid, the other rests upon the dressings, or is suspended above them. The water follows the band of cloth by virtue of capillary attraction, or after the manner of a syphon. For the purpose of draining off the water, the box has a concave bottom, pierced with large holes, through which the liquid may escape underneath; from whence a tube conducts it into another vessel placed without the bed. The member rests in the box upon a soft cushion, covered with an impermeable cloth."

*Apparatus of M. Equisier.*

This apparatus is composed, as we know, of the body of a pump in which moves a heavy lead piston. At the lower extremity of the reservoir is an orifice furnished with a spigot, to which also is attached an elastic tube designed to conduct the water; the liquid is forced out by the weight of the piston. This apparatus is very convenient because it can be placed upon any piece of furniture near the bed of the patient; but in general it is too expensive to be much employed, if it is of a proper and convenient size. Those ordinarily used are very small and necessarily require to be refilled constantly The same is true of all those numerous different forms of apparatus in which the liquid is forced out by a piston put in motion by galvanized caoutchouc tubes or strips.

Our own apparatus and method of irrigation is as follows:

A sufficiently large vessel, of zinc or earthenware, is placed upon an elevated

* The translator has taken great liberty with this paragraph on account of the careless, or perhaps he ought rather to say, *idiomatic,* manner in which the description is given in the original. Similar liberties have occasionally been taken elsewhere, when the correct understanding of the text seemed to demand it.—T.

piece of furniture, near the bed; an elastic syphon, furnished with a stop-cock, guides the water from the reservoir upon the tissues. The part undergoing irrigation, is quite insulated by the aid of a gutter, or of impermeable cloth so arranged as that the water does not moisten the neighboring parts, and escapes freely into a vessel placed near.

A compress placed upon the member, serves to disperse the water uniformly. Ordinarily we adapt to the extremity of the syphon tubes, a strip of linen divided into bandalets which serve not only to direct the liquid, but principally to obviate its dynamic effect.

Once mounted, this apparatus works of itself, provided that care is taken to prevent the vessel from becoming empty, so that the flow of water may not be intermittent. We ought also to be particular in relation to the cleanliness and especially the temperature of the water, which should always be uniform, and not cause any disagreeable sensation to the patient.

When we wish to submit an extensive surface to continued and abundant irrigation, we ought to employ an apparatus which discharges its water in several streams, rather than one which is furnished with a single large conductor. In fact, when the water flows in a large stream it becomes warmer as it passes over the tissues, and the whole surface is not submitted to a uniform temperature.

If continued irrigations are to be made in any case with cold or ice water, it will be best to commence with tepid water, and gradually lower the temperature. Without this precaution, we are in danger, not only of causing to the patient very disagreeable sensations, but even grave accidents. In a case of extensive burn, Sanson saw tetanus supervene in consequence of the application of very cold water.

In pursuance of the principle which we have sought to establish, we ought to be exceedingly careful that the temperature of the water is not suddenly changed, and especially that its current is not interrupted, for a severe inflammatory reaction might result. When it is thought proper to suspend the water, its temperature should be gradually raised, and we ought even, as Cloquet advises, apply it for only a few hours each day before withholding it completely.

The quantity of water, it appears to us, should be regulated by the degree of inflammation, that is to say, if it is feeble a continued moisture will suffice; if very intense, on the contrary, the fluid should be poured upon it very rapidly, inundating, so to speak, the part, especially if the water used has a temperature of 18°.or 20° C. (64° or 68° F.) This liquid placed in contact with the tissues subtracts morbid caloric, and the more there is developed, the more water should be poured on, taking care, however, that the temperature is not too much below the standard of health.

The irrigation ought to be made, not only upon the part wounded, but also upon a considerable extent of surface about; without this precaution, serious accidents may occur, in parts which we supposed to be at first sound, and when no inflammation is developed at the point of injury, which is submitted to the action of the water.

I possess many cases which prove clearly, that persons wounded have succumbed from accidents developed in the vicinity of the point where the irrigation was made, and this doubtless explains why some authors have said that water masks the phenomena of inflammation, and does not prevent the development of purulent channels. I do not deny that this may happen, even when irrigation is well applied; but I am persuaded that in most cases the cause will be found in the application of the water to a too limited surface.

The absence of any of those materials of which I have spoken, often compels the surgeon to modify his apparatus; I shall, therefore, notice hastily the means which will be found most convenient in such an emergency.

For the upper receptacle we may use almost any vessel, but especially a pail of zinc or of wood, such as may be found almost everywhere.

In order to have the vessel above the part to be irrigated, we can, if it is a pail, suspend it to a nail in the wall, or place it upon a chair elevated upon a table. My father often uses a double ladder, upon the rounds of which he places a board, and on that the vessel. The pail may be also suspended from a strong pole sustained in the same way as the board.

In the country we find quite often, bedsteads with upright posts, to which we may secure a cross piece, which will answer the purpose. Two high tables, one being placed on each side of the bed, may be used instead of the posts. In short, we can in an emergency make irrigations with a simple watering pot.

In civil hospitals, the upper vessel, usually a pail, is suspended from the horizontal iron cross bar, which supports also the chord with which the patient aids himself in his movements. I could mention other modes, but these will suffice.

To the lower part of the vessel we may attach a small stop cock, and to this a strip of linen, which will serve to direct the stream of water: a small common fountain would answer the purpose also well enough.

*Temperatare most suitable for irrigations.*—Upon this subject I will only add to what I have already said when speaking of temperature generally, that cold irrigations have almost always been employed by MM. A. Josse, Berard, and Breschet, and that after having experienced some of the inconveniences of cold they have concluded that we ought to be governed by the sensations of the patient in determining upon the degree of heat or cold.

According to our own experience, tepid water is preferable in a majority of cases; yet it is wrong to proscribe cold water absolutely, as we have before said.

*How long the irrigation ought to be continued.*—It is difficult to fix the proper duration of irrigations, for it must depend upon the nature of the affection, the temperament of the patient, the stage, whether acute or chronic, of the malady. Nevertheless I will state some of the rules which surgeons most accustomed to the use of irrigations, have laid down.

Those who employ cold or ice water, are in the habit of suspending it when the patient complains of a feeling of cold in the part submitted to its action; but we must not be too hasty, for M. Josse tells us "that under these circumstances he had but too often seen the inflammation reproduced in all its former intensity;" and we have ourselves often verified the justness of this remark. It will be advisable then, not to suspend the application of the water, but gradually to raise its temperature until the patient no longer experiences any disagreeable sensation, and not to change the treatment until all chance of inflammation has disappeared. When we use tepid water we may continue the irrigation a much longer time, since the process of reparation of traumatic lesions goes on very regularly under its influence. My father employs them ordinarily fifteen, twenty, or twenty-five days, and then he substitutes for them water dressings which he renews as often as may be necessary to preserve their moisture. We ought, however, to resume the irrigations when a return of inflammation is threatened.

I cannot here indicate all the cases in which irrigations might be employed. I will simply say that they can be advantageously employed in the most grave surgical accidents, in all violent inflammations which are with difficulty moderated or controlled by ordinary means, and also in severe wounds which secrete pus copiously.

After what I have said under the general considerations, I shall not dwell long upon the advantages of irrigations, and the inconveniences which have been ascirибed to them.

*Advantages.*—In violent traumatic inflammations, water constantly renewed and kept at a uniform temperature, abstracts the morbid caloric, and afterwards, when supuration is established, the pus is removed as fast as it is produced, and we have less reason to apprehend purulent absorption and its consequences.

*Inconveniencies.*—In truth I know of none which may not be attributed to any other therapeutic agent if improperly applied.

If, for example, the temperature is too low, there may result a too violent repercussion of inflammation, gangrene, &c. If the water does not escape freely, but only irregularly, there will follow mischievous alternations of action and reaction, the same as if the temperature was irregular.

These points demand, therefore, the greatest attention.

It has also been said, that irrigations act superficially. The numerous cases of grave and deep lesions, in which this means has been employed with wonderful success, do not permit me to hold this opinion.

*En resume*, continued irrigation is a powerful means which has already rendered the greatest service in many cases, and which could render still greater service if it were better appreciated. I shall be happy if I have succeeded in recalling attention to this means, and if I have successfully shown that the inconveniences with which it has been charged, are due mostly to the too low temperature of the liquid employed. On account of the difficulty of finding an apparatus suited to all circumstances in which we may be placed, I have sought to indicate simple methods, and such as may be adopted everywhere. If, however, irrigations could not be employed, we might have recourse to immersions, or to the water-dressings.

### IMMERSION.

Immersion is the act of plunging the body, or some portion of it, into a liquid; in a word, it is a bath, either general or local. In surgery, immersions being usually made during a certain period, and with a uniform temperature, and sometimes even with constant renewal of the liquid, it is proper, I think, to speak of the immersion as *continued*, as we do of irrigation. Perhaps, also, the word *maceration* might be properly substituted for immersion.

The surgical employment of immersions dates back, I believe, to Lamorier, as our readers can have seen in the historical part of this work. The three cases which he cites are sufficiently remarkable, and had I chosen to speak of those in which he employed mineral waters, I might have referred to others.

"There are," says Percy, "external affections in which the local heat is so intense as to dry rapidly the thickest compresses drenched with water. We risk nothing, then, in applying them *moderately cold;* and if the part can be

*plunged in a bath*, nothing will more effectually check the fury of the vital actions, and restore calm and regularity to the orgasm."

Since Percy, immersions had been employed but seldom, except by the military surgeons, especially in cases of sprains, when in 1841, M. Charles Mayor published a memoir, entitled, *On the Localization of Baths upon the different parts of the human body.* In this very interesting work, the surgeon of Lausanne has endeavored to call the attention of surgeons to the advantages which can be derived from the use of water by immersion, and to make known the various kinds of apparatus which he has invented for the purpose of applying insulated baths to different portions of the body.

The apparatus of M. C. Mayor consists of metallic vessels adapted to the form and size of the part which they are destined to receive; they are terminated in *cul de sacs* if designed to bathe the extremities of the limbs, and open at both ends if intended for other parts, and furnished also with ruffles made of caoutchouc, or bladder, to prevent the water from escaping.

M. C. Mayor closes his memoir with the following conclusions:

"First — These different kinds of apparatus are easily obtained in any place, and at a small cost.

"Second — They enable us to localize a bath upon all portions of the extremities, and without the patient being obliged to put his limb out of the bed, or in a declining position, which is often fatiguing and sometimes mischievous or impossible; we ought always to be able to select and keep that position which is most convenient.

"Third — The liquid not undergoing any evaporation, will retain its warmth a long time, especially if we take the precaution to surround the vessel with a bad conductor of caloric. The slight subtraction of caloric resulting from the coolness of the walls of the vessel, will be compensated by the heat which the diseased limb supplies to the small quantity of water surrounding it.

"Fourth — If we wish to bathe the hand, the forearm, or the arm, the apparatus furnished with rings and cords, might, under certain circumstances, be suspended to the neck of the patient by means of a sling, and he could thus be permitted to go out and attend to his business while he is at the same time continuing his bath.

"Fifth — The liquid can be renewed without changing the position of the limb, or cooling the part which is subjected to the bath.

"Sixth — Since it is necessary to use but little liquid, the cost of combustibles is almost nothing, and the trouble of preparing it is trivial; and if medicated baths are employed, they will also be attended with but little expense, ven when such expensive articles as the iodurets, the sublimates, &c., are employed.

"Seventh — The vessel being hermetically closed, does not permit any of the volatile principle to escape which the water may contain.

"Eighth — The apparatus having two openings, can be used where we wish to submit a limb, or a portion of a limb, to a continued current of water. For this purpose we have only to adapt to the upper orifice an elastic tube communicating with a vessel placed at a height proportionate to the de-degree of impulsion we wish to give to the water. The inferior remains open, or it may be made smaller by inserting into it a perforated cork, or a stop-cock."

Our learned colleague, Dr. Lebert, who has employed local baths with the apparatus of M. C. Mayor, says: "I have used these baths in a great number of cases, and found them most useful in the diseases of joints. In part as the result of experience, and in part by a natural argument, I have deduced the following therapeutical indications for their employment:

"If the integuments are intact in acute or sub-acute inflammatory affections, as for example in case of felons, inflammations of the ligaments or fibrous structures, either of the feet, hands or knees, etc., and in general wherever there is severe pain and tension, we ought to employ them to sooth, or even to encourage the bleeding from leeches. These baths generally preserve an equal temperature for a considerable length of time, and possess also the great advantage of keeping the parts from contact with the air. But we ought not to continue them more than an hour consecutively, as the longer confinement will incommode the patient. We have had occasion to observe the soothing effect of local baths, with the water of Lavey alone, in many cases of acute inflammation, and of abscesses occurring during the treatment of articular diseases, white swelling and caries. * * * It has seemed to me that the absorption of the water of the bath, in these cases, was greater in local baths almost hermetically closed, than in those taken by the ordinary method. In eight cases of diseases of the bones, I have seen the suppuration promptly diminished, and the condition of the soft parts improved, by this treatment. In several cases of white swelling and of caries of the foot, the patients could not endure the position requisite for the ordinary foot bath, while they have taken the local baths with the apparatus of Dr. Mayor, without changing their position, and while lying in bed."

Immersions, or local baths, applicable chiefly to the extremities of the limbs, are destined, in certain cases, to render great service to surgery, and to be of frequent use, owing to the ease with which they can be procured and administered. This method of applying water is exceedingly simple for lesions of the forearm or hand, and for the leg or foot. It is only necessary to place near the patient a vessel containing water of a mild and agreeable temperature, into which the part may be completely plunged. Care must be taken that the temperature of the liquid does not sensibly vary, and that it is renewed sufficiently often to keep it clean. For the arm, thigh, and pelvis, we may employ the very ingenious apparatus of M. Charles Mayor, junior, not forgetting, however, that their protracted use may be attended with inconveniences by the constriction which they produce above the diseased part. * * *

In order to determine the variations of temperature of the water, so that it may be renewed at the proper moment, we should use a thermometer; and for the greater convenience we may adapt two tubes to the bath, the one for the admission and the other for the escape of the fluid.

As to the position of the part undergoing an immersion, we can only say that we must always have regard to the escape of the pus and the return of the venous blood.

The good effects of prolonged immersions in water of a mild temperature, appear to me to be attributable, as in irrigations, to the subtraction of caloric, and also without doubt, to the absorption of the liquid, which penetrates the tissues. * * *

Water at 18° or 20° C (64° or 68° F) more or less, is generally preferable to cold water, and it is at this temperature that my father always employs it in his practice.

As to the duration of the immersions, it must vary much, according to the degree, extent, and depth of the inflammation, and it is impossible to establish precise rules; nevertheless, I think they ought to be prolonged sufficiently to insure against a return of the inflammation, and then they should be discontinued gradually, replacing them at first with water dressings.

To avoid the inconvenience of absolute confinement of the patient, we may in certain cases, as in lesions of the fingers and penis for example, use a pig's bladder or a caoutchouc bag, filled with water, into which the part may be immersed and secured.

In the most severe surgical accidents, we should commence, if possible, with immersions or irrigations, or both, and terminate with water dressings. Sometimes even we may unite the three modes for the purpose of avoiding the usual painful dressings, and securing those great benefits which we have a right to expect from water.

Finally, immersion is a procedure which seems to us to have great efficacy in cases where the water is required to act upon the deep structures: here, therefore, it can be profitably substituted for irrigations, whose effects are in general more superficial.

In comparing the three principal modes, we observe that the water dressing, if properly made, and often renewed, is generally more simple, and more easily applied, than immersion or irrigation, that it has, moreover, the advantage of taking off at each dressing the pus and the stratum formed by a mixture of the purulent matter and the substances contained in the water; the granulations are thus kept always red, &c.

Irrigation is more powerful than water dressing, but more difficult, and sometimes even impossible to apply.

Immersion is more powerful than either, more prompt, and in all respects superior whenever it can be properly applied.

### CONCLUSIONS.

The history of this branch of surgical therapeutics, proves that from the origin of the art of surgery, and even before this epoch, water was the agent to which recourse was instinctively had to assuage pain.

But soon miraculous waters, balsams, and ointments of all kinds, were employed, and their use was continued during a long succession of ages under the protection of barbarism and charlatanism.

At last that great school, the Academy of Surgery, did justice to polypharmacy, and directed all its efforts to simplify dressings; but it was only at rare intervals that a surgeon recognized the superiority of water for the accomplishment of this end.

Latterly, efforts have been made to demonstrate the utility of water: but hitherto the old methods have prevailed, irrigations only being employed, and even their use is very limited, and so to speak, exceptional.

Water used locally, at a proper temperature, is, in the treatment of surgical affections, the most powerful antiphlogistic which we possess; it is also the most easily procured and the most easily applied; it cleanses, cools the wound, and assuages the pain; it is, in short, the best balm, and, as Briot says, *le vulneraire par excellence.*

The use of water topically, embraces three principal modes under which all others may be classed; these are water-dressings, irrigation, and immersion.

Water-dressings, such as we have described, that is, properly moistened, and renewed, are destined to render important services to surgery.

Irrigation is an excellent means, which already has rendered much service; but it is too seldom employed, and, indeed, it has been almost abandoned, on account of the difficulty attending its application, and the accidents resulting from the too low temperature at which it has been generally employed.

We have sought to diminish its inconveniences by indicating more simple apparatus, and by insisting upon the use of tepid water, which it appears to us, ought to be preferred in the great majority of cases; still we think that immersion and water-dressings can often be substituted with advantage.

Immersion, or the local bath, more or less prolonged, is too much neglec-ted, even where the parts can be most easily immersed; it is a means whose results are very prompt and infinitely superior to cataplasms, and even to irrigations or water-dressings.

By these three methods, separate, or combined, we can meet every indication and obtain results truly wonderful. It is sufficient to make trial of them in lesions of the fingers and penis, to force a conviction of their superiority over all other modes of dressing, and to ensure their application afterwards, in the most serious lesions, wherever their seat.

Finally, I have endeavored in this essay by historical documents, and by comparative experiments, to establish the advantages of water over every other topical application; and after the remarkable results which have already been obtained, I am encouraged to hope that soon this therapeutical agent will occupy the first place in surgery as a local antiphlogistic.

